# Genome sequence of the South American clover-nodulating *Rhizobium leguminosarum* bv. *trifolii* strain WSM597

**DOI:** 10.4056/sigs.4508258

**Published:** 2013-12-15

**Authors:** Wayne Reeve, Jason Terpolilli, Vanessa Melino, Julie Ardley, Rui Tian, Sofie De Meyer, Ravi Tiwari, Ronald Yates, Graham O’Hara, John Howieson, Mohamed Ninawi, Brittany Held, David Bruce, Chris Detter, Roxanne Tapia, Cliff Han, Chia-Lin Wei, Marcel Huntemann, James Han, I-Min Chen, Konstantinos Mavromatis, Victor Markowitz, Natalia Ivanova, Galina Ovchinnikova, Ioanna Pagani, Amrita Pati, Lynne Goodwin, Tanja Woyke, Nikos Kyrpides

**Affiliations:** 1 Centre for Rhizobium Studies, Murdoch University, Western Australia, Australia; 2 Department of Agriculture and Food, Western Australia, Australia; 3 DOE Joint Genome Institute, Walnut Creek, California, USA; 4 Los Alamos National Laboratory, Bioscience Division, Los Alamos, New Mexico, USA; 5 Biological Data Management and Technology Center, Lawrence Berkeley National Laboratory, Berkeley, California, USA

**Keywords:** root-nodule bacteria, nitrogen fixation, rhizobia, *Alphaproteobacteria*

## Abstract

*Rhizobium leguminosarum* bv. *trifolii* strain WSM597 is an aerobic, motile, Gram-negative, non-spore-forming rod isolated from a root nodule of the annual clover *Trifolium pallidum* L. growing at Glencoe Research Station near Tacuarembó, Uruguay. This strain is generally ineffective for nitrogen (N_2_) fixation with clovers of Mediterranean, North American and African origin, but is effective on the South American perennial clover *T. polymorphum* Poir. Here we describe the features of *R. leguminosarum* bv. *trifolii* strain WSM597, together with genome sequence information and annotation. The 7,634,384 bp high-quality-draft genome is arranged in 2 scaffolds of 53 contigs, contains 7,394 protein-coding genes and 87 RNA-only encoding genes, and is one of 20 rhizobial genomes sequenced as part of the DOE Joint Genome Institute 2010 Community Sequencing Program.

## Introduction

A key factor which limits the productivity of agricultural systems is the availability of soil nitrogen (N). Legumes can overcome soil N limitations by forming symbiotic relationships with root nodule bacteria (rhizobia). Rhizobia, through their interaction with legumes, are able to reduce atmospheric dinitrogen (N_2_) into ammonia, which can supply essential N for growth to the plant. In addition, much of this fixed N is subsequently released into the soil following plant senescence and decay, grazing by livestock or human harvest [1], thereby increasing soil N content and fertility for subsequent crops. Thus, biological N_2_ fixation forms a vital component of sustainable agriculture as it provides a means of ameliorating N-deficient soils without the need for industrially synthesized N-based fertilizers, the production and application of which have significant environmental and economic costs [2].

Forage and fodder legumes play an integral role in sustainable farming practice, providing feed for stock while also enriching soil with available N. Worldwide, there are approximately 110 million ha of forage and fodder legumes under production [3], of which *Trifolium* spp. (clover) are of key importance [4]. The bacterial microsymbionts that nodulate clovers are *Rhizobium leguminosarum* bv. *trifolii*. Since *Trifolium* spp. are geographically widely distributed and are also phenologically variable (i.e. they may be either annual [e.g. *T. subterraneum, T. pallidum and T. scutatum*] or perennial [e.g. *T. pratense, T. repens* and *T. polymorphum*]), it is rare that a single strain of *R. leguminosarum* bv. *trifolii* can effectively fix N_2_ across a wide diversity of clovers [5].

*Rhizobium leguminosarum* bv. *trifolii* strain WSM597 was isolated from the nodules of *Trifolium pallidum*, which were collected from the INIA Glencoe Research Station, Uruguay in 1999. WSM597 is able to nodulate (Nod^+^) and fix (Fix^+^) N_2_ effectively on the South American perennial clover *Trifolium polymorphum*. However, while WSM597 is able to nodulate *Trifolium pallidum* and other annual and perennial *Trifolium* spp. of Mediterranean, African and North American origin, it is not effective for N_2_ fixation on any of these hosts (Yates *et al.,* unpublished data). Therefore, WSM597 is highly specific for effectiveness in symbiosis, as is also evident with the recently sequenced South American clover microsymbiont *R. leguminosarum* bv. *trifolii* WSM2304 [6]. Thus, both microsymbionts demonstrate that phenological and geographic barriers exist for effective nodulation in clover symbioses. As this phenotype represents a common challenge to managing the legume-rhizobial symbiosis in agriculture, the genome of WSM597 is a valuable comparator for genetic studies of nodulation and N_2_ fixation. Here we present a summary classification and a set of general features for *R. leguminosarum* bv. *trifolii* strain WSM597 together with a description of the genome sequence and annotation.

## Classification and general features

**Figure 1:**
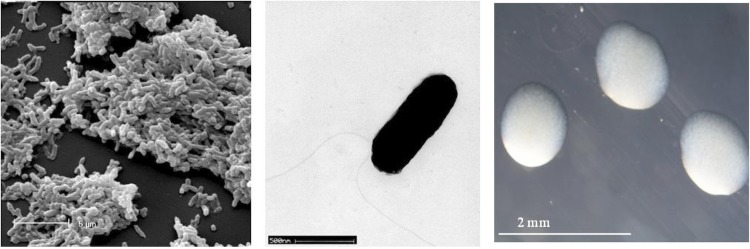
Images of *Rhizobium leguminosarum* bv. *trifolii* strain WSM597 using scanning (Left) and transmission (Center) electron microscopy as well as light microscopy to visualize colony morphology on a solid medium (Right).

**Figure 2:**
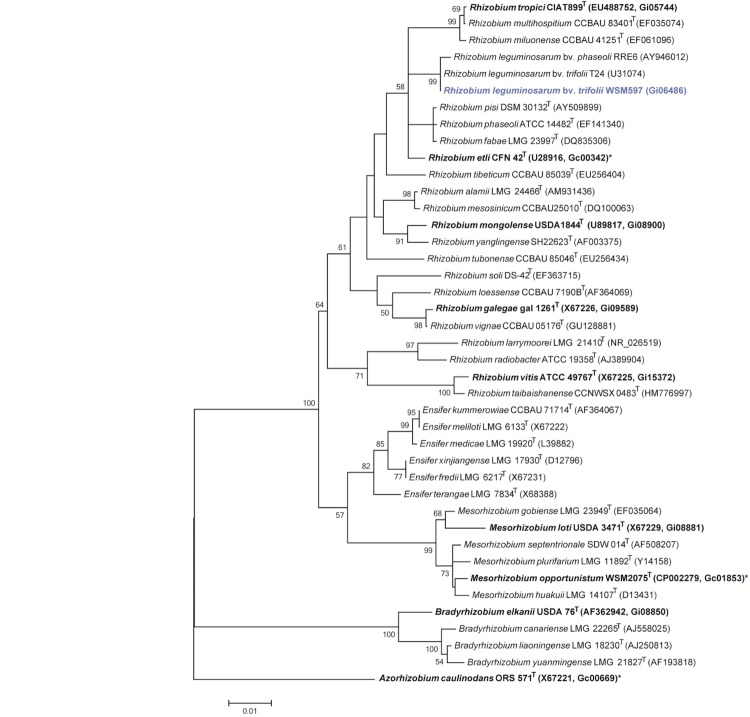
Phylogenetic tree showing the relationships of *Rhizobium leguminosarum* bv. *trifolii* strain WSM597 (shown in blue print) with some of the root nodule bacteria in the order *Rhizobiales* based on aligned sequences of the 16S rRNA gene (1,307 bp internal region). All sites were informative and there were no gap-containing sites. Phylogenetic analyses were performed using MEGA, version 5.05 [23]. The tree was built using the maximum likelihood method with the General Time Reversible model. Bootstrap analysis [24] with 500 replicates was performed to assess the support of the clusters. Type strains are indicated with a superscript T. Strains with a genome sequencing project registered in GOLD [25] are in bold print and the GOLD ID is mentioned after the accession number. Published genomes are designated with an asterisk.

*R. leguminosarum* bv. *trifolii* strain WSM597 is a motile, Gram-negative rod (Figure Left and Center) in the order *Rhizobiales* of the class *Alphaproteobacteria*. It is fast growing in laboratory culture, forming colonies within 3-4 days when grown on half Lupin Agar (½LA) [7] at 28°C. Colonies on ½LA are white-opaque, slightly domed, moderately mucoid with smooth margins (Figure 1 Right). Minimum Information about the Genome Sequence (MIGS) is provided in Table 1. Figure 2 shows the phylogenetic neighborhood of *R. leguminosarum* bv. *trifolii* strain WSM597 in a 16S rRNA sequence based tree. This strain clusters closest to *Rhizobium leguminosarum* bv. *trifolii* T24 and *Rhizobium leguminosarum* bv. *phaseoli* RRE6 with 99.9% and 99.8% sequence identity, respectively.

**Table 1:** Classification and general features of *Rhizobium leguminosarum* bv. *trifolii* strain WSM597 according to the MIGS recommendations [8].

**MIGS ID**	**Property**	**Term**	**Evidence code**
	Current classification	Domain *Bacteria*	TAS [9]
		Phylum *Proteobacteria*	TAS [10]
		Class *Alphaproteobacteria*	TAS [11,12]
		Order *Rhizobiales*	TAS [12,13]
		Family *Rhizobiaceae*	TAS [14,15]
		Genus *Rhizobium*	TAS [14,16-19]
		Species *Rhizobium leguminosarum* bv. *trifolii*	IDA [14,16,19,20]
	
	Gram stain	Negative	IDA
	Cell shape	Rod	IDA
	Motility	Motile	IDA
	Sporulation	Non-sporulating	NAS
	Temperature range	Mesophile	NAS
	Optimum temperature	28°C	NAS
MIGS-22	Oxygen requirement	Aerobic	NAS
	Carbon source	Varied	IDA
	Energy source	Chemoorganotroph	NAS
MIGS-6	Habitat	Soil, root nodule on host	IDA
MIGS-15	Biotic relationship	Free living, symbiotic	IDA
MIGS-14	Pathogenicity	Non-pathogenic	NAS
	Biosafety level	1	TAS [21]
	Isolation	Legume root nodule	IDA
MIGS-4	Geographic location	Tacuarembó, Uruguay	IDA
MIGS-5	Nodule collection date	1999	IDA
MIGS-4.1	Longitude	-56	IDA
MIGS-4.2	Latitude	-31.41	
MIGS-4.3	Depth	5 cm soil depth	
MIGS-4.4	Altitude	130 m	

Evidence codes – IDA: Inferred from Direct Assay (i.e. first time published); TAS: Traceable Author Statement (i.e., a direct report exists in the literature) NAS: Non-traceable Author Statement (i.e., not directly observed for the living, isolated sample, but based on a generally accepted property for the species, or anecdotal evidence). These evidence codes are from the Gene Ontology project [22].

### Symbiotaxonomy

*R. leguminosarum* bv. *trifolii* WSM597 nodulates (Nod^+^) and fixes N_2_ effectively (Fix^+^) with the South American perennial clover *T. polymorphum*. However, WSM597 is ineffective on perennial clovers of North American (*T. reflexum* and *T. amabile*) and African origin (*T. sempilsoum*). WSM597 is also ineffective on a range of Mediterranean annuals (*T. resupinatum, T. clusii, T. michelianum, T. isthmocarpum, T. scutatum, T. incarnatum, T. tomentosum*), including its host of origin *T. pallidum* and the North American annual *T. bejariense* (Yates, R., pers. comm.).

## Genome sequencing and annotation information

### Genome project history

This organism was selected for sequencing on the basis of its environmental and agricultural relevance to issues in global carbon cycling, alternative energy production, and biogeochemical importance, and is part of the Community Sequencing Program at the U.S. Department of Energy, Joint Genome Institute (JGI) for projects of relevance to agency missions. The genome project is deposited in the Genomes OnLine Database [25] and an improved-high-quality-draft genome sequence in IMG. Sequencing, finishing and annotation were performed by the JGI. A summary of the project information is shown in Table 2.

**Table 2:** Genome sequencing project information for *Rhizobium leguminosarum* bv. *trifolii* strain WSM597.

**MIGS ID**	**Property**	**Term**
MIGS-31	Finishing quality	Improved high-quality draft
MIGS-28	Libraries used	Illumina GAii shotgun and paired end 454 libraries
MIGS-29	Sequencing platforms	Illumina GAii and 454 GS FLX Titanium technologies
MIGS-31.2	Sequencing coverage	7.8× 454 paired end, 764.2× Illumina
MIGS-30	Assemblers	Velvet 1.0.13, Newbler 2.3, phrap 4.24
MIGS-32	Gene calling methods	Prodigal 1.4, GenePRIMP
	GOLD ID	Gi06486
	NCBI project ID	65299
	Database: IMG	2509276021
	Project relevance	Symbiotic N_2_ fixation, agriculture

## Growth conditions and DNA isolation

*Rhizobium leguminosarum* bv. *trifolii* strain WSM597 was grown to mid logarithmic phase in TY rich medium [26] on a gyratory shaker at 28°C. DNA was isolated from 60 mL of cells using a CTAB (Cetyl trimethyl ammonium bromide) bacterial genomic DNA isolation method [27].

### Genome sequencing and assembly

The genome of *Rhizobium leguminosarum* bv. *trifolii* strain WSM597 was sequenced at the Joint Genome Institute (JGI) using a combination of Illumina [28] and 454 technologies [29]. An Illumina GAii shotgun library which generated 73,610,574 reads totaling 5,594.4 Mb, and a paired end 454 library with an average insert size of 14 Kb which generated 335,966 reads totaling 93.4 Mb of 454 data were generated for this genome. All general aspects of library construction and sequencing performed at the JGI can be found at the JGI website [30]. The initial draft assembly contained 190 contigs in 6 scaffolds. The 454 Titanium standard data and the 454 paired end data were assembled together with Newbler, version 2.3-PreRelease-6/30/2009. The Newbler consensus sequences were computationally shredded into 2 Kb overlapping fake reads (shreds). Illumina sequencing data were assembled with VELVET, version 1.0.13 [31], and the consensus sequences were computationally shredded into 1.5 Kb overlapping fake reads (shreds). The 454 Newbler consensus shreds, the Illumina VELVET consensus shreds and the read pairs in the 454 paired end library were integrated using parallel phrap, version SPS - 4.24 (High Performance Software, LLC). The software Consed (Ewing and Green 1998; Ewing et al. 1998; Gordon et al. 1998) was used in the following finishing process. Illumina data was used to correct potential base errors and increase consensus quality using the software Polisher developed at JGI (Alla Lapidus, unpublished). Possible mis-assemblies were corrected using gapResolution (Cliff Han, unpublished), Dupfinisher (Han, 2006), or sequencing cloned bridging PCR fragments with subcloning. Gaps between contigs were closed by editing in Consed, by PCR and by Bubble PCR (J-F Cheng, unpublished) primer walks. A total of 215 additional reactions were necessary to close gaps and to raise the quality of the finished sequence. The estimated genome size is 7.3 Mb and the final assembly is based on 57.2 Mb of 454 draft data which provides an average 7.8× coverage of the genome and 5,578.3 Mb of Illumina draft data which provides an average 764.2× coverage of the genome.

### Genome annotation

Genes were identified using Prodigal [32] as part of the DOE-JGI Annotation pipeline [33], followed by a round of manual curation using the JGI GenePRIMP pipeline [34]. The predicted CDSs were translated and used to search the National Center for Biotechnology Information (NCBI) non-redundant database, UniProt, TIGRFam, Pfam, PRIAM, KEGG, COG, and InterPro databases. These data sources were combined to assert a product description for each predicted protein. Non-coding genes and miscellaneous features were predicted using tRNAscan-SE [35], RNAMMer [36], Rfam [37], TMHMM [38], and SignalP [39]. Additional gene prediction analyses and functional annotation were performed within the Integrated Microbial Genomes (IMG-ER) platform [40].

## Genome properties

**Figure 3:**
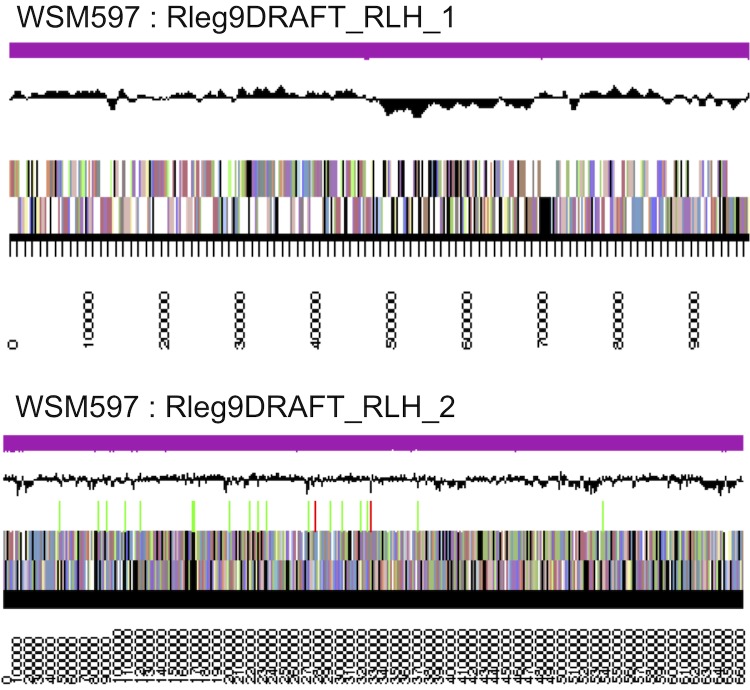
Graphical map of the two DNA scaffolds of *Rhizobium leguminosarum* bv. *trifolii* strain WSM597. From outside to the center: Genes on forward strand (color by COG categories as denoted by the IMG platform), Genes on reverse strand (color by COG categories), RNA genes (tRNAs green, sRNAs red, other RNAs black), GC content, GC skew.

The genome is 7,634,384 nucleotides with 61.01% GC content (Table 3) in 2 scaffolds containing 53 contigs. From a total of 7,481 genes, 7,394 were protein encoding and 87 RNA only encoding genes. The majority of genes (79.24%) were assigned a putative function whilst the remaining genes were annotated as hypothetical. The distribution of genes into COGs functional categories is presented in Table 4 and Figure 3.

**Table 3:** Genome Statistics for *Rhizobium leguminosarum* bv. *trifolii* strain WSM597.

**Attribute**	**Value**	**% of Total**
Genome size (bp)	7,634,384	100.00
DNA coding region (bp)	6,596,806	86.41
DNA G+C content (bp)	4,657,890	61.01
Number of scaffolds	2	
Number of contigs	53	
Total genes	7,481	100.00
RNA genes	87	1.16
rRNA operons*	1	
Protein-coding genes	7,394	98.84
Genes with function prediction	5,928	79.24
Genes assigned to COGs	5,886	78.68
Genes assigned Pfam domains	6,150	82.21
Genes with signal peptides	634	8.47
Genes with transmembrane helices	1,655	22.12
CRISPR repeats	0	

*1 extra 5s rRNA and 2 extra 16s rRNA genes

**Table 4:** Number of protein coding genes of *Rhizobium leguminosarum* bv. *trifolii* strain WSM597 associated with the general COG functional categories.

**Code**	**Value**	**%age**	**Description**
J	195	2.95	Translation, ribosomal structure and biogenesis
A	0	0.00	RNA processing and modification
K	627	9.50	Transcription
L	233	3.53	Replication, recombination and repair
B	2	0.03	Chromatin structure and dynamics
D	44	0.67	Cell cycle control, mitosis and meiosis
Y	0	0.00	Nuclear structure
V	73	1.11	Defense mechanisms
T	375	5.68	Signal transduction mechanisms
M	333	5.05	Cell wall/membrane biogenesis
N	108	1.64	Cell motility
Z	1	0.02	Cytoskeleton
W	0	0.00	Extracellular structures
U	107	1.62	Intracellular trafficking and secretion
O	200	3.03	Posttranslational modification, protein turnover, chaperones
C	351	5.32	Energy production conversion
G	674	10.21	Carbohydrate transport and metabolism
E	748	11.33	Amino acid transport metabolism
F	109	1.65	Nucleotide transport and metabolism
H	211	3.20	Coenzyme transport and metabolism
I	242	3.67	Lipid transport and metabolism
P	297	4.50	Inorganic ion transport and metabolism
Q	171	2.59	Secondary metabolite biosynthesis, transport and catabolism
R	850	12.88	General function prediction only
S	649	9.83	Function unknown
-	1,595	21.32	Not in COGS

## References

[1] Howieson JG O’Hara GW Carr SJ Changing roles for legumes in Mediterranean agriculture: developments from an Australian perspective. Field Crops Res 2000; 65:107-122 10.1016/S0378-4290(99)00081-7

[2] Good AG Beatty PH Fertilizing nature: a tragedy of excess in the commons. PLoS Biol 2011; 9:e1001124 10.1371/journal.pbio.1001124 21857803 PMC3156687

[3] Herridge DF Peoples MB Boddey RM Global inputs of biological nitrogen fixation in agricultural systems. Plant Soil 2008; 311:1-18 10.1007/s11104-008-9668-3

[4] Zohary M, Heller D. The Genus *Trifolium* Jerusalem: The Israel Academy of Sciences and Humanities Ahva Printing Press; 1984.

[5] Howieson J Yates R O'Hara G Ryder M Real D The interactions of *Rhizobium leguminosarum* biovar *trifolii* in nodulation of annual and perennial *Trifolium* spp. from diverse centres of origin. Aust J Exp Agric 2005; 45:199-207 10.1071/EA03167

[6] Reeve W O'Hara G Chain P Ardley J Brau L Nandesena K Tiwari R Malfatti S Kiss H Lapidus A Complete genome sequence of *Rhizobium leguminosarum* bv. *trifolii* strain WSM2304, an effective microsymbiont of the South American clover *Trifolium polymorphum.* Stand Genomic Sci 2010; 2:66-76 10.4056/sigs.44642 21304679 PMC3035254

[7] Howieson JG Ewing MA D'antuono MF Selection for acid tolerance in *Rhizobium meliloti.* Plant Soil 1988; 105:179-188 10.1007/BF02376781

[8] Field D Garrity G Gray T Morrison N Selengut J Sterk P Tatusova T Thomson N Allen M Angiuoli SV Towards a richer description of our complete collection of genomes and metagenomes "Minimum Information about a Genome Sequence " (MIGS) specification. Nat Biotechnol 2008; 26:541-547 10.1038/nbt1360 18464787 PMC2409278

[9] Woese CR Kandler O Wheelis ML Towards a natural system of organisms: proposal for the domains *Archaea, Bacteria*, and *Eucarya.* Proc Natl Acad Sci USA 1990; 87:4576-4579 10.1073/pnas.87.12.4576 2112744 PMC54159

[10] Garrity GM, Bell JA, Lilburn T. Phylum XIV. *Proteobacteria* phyl. nov. In: Garrity GM, Brenner DJ, Krieg NR, Staley JT (eds), Bergey's Manual of Systematic Bacteriology, Second Edition, Volume 2, Part B, Springer, New York, 2005, p. 1.

[11] Garrity GM, Bell JA, Lilburn T. Class I. *Alphaproteobacteria* class. In: Garrity GM, Brenner DJ, Kreig NR, Staley JT, editors. Bergey's Manual of Systematic Bacteriology. Second ed: New York: Springer - Verlag; 2005, p. 1.

[12] Validation List No 107. List of new names and new combinations previously effectively, but not validly, published. Int J Syst Evol Microbiol 2006; 56:1-6 10.1099/ijs.0.64188-0 16403855

[13] Kuykendall LD. Order VI. *Rhizobiales* ord. nov. In: Garrity GM, Brenner DJ, Kreig NR, Staley JT, editors. Bergey's Manual of Systematic Bacteriology. Second ed: New York: Springer - Verlag; 2005. p 324.

[14] Skerman VBD McGowan V Sneath PHA Approved Lists of Bacterial Names. Int J Syst Bacteriol 1980; 30:225-420 10.1099/00207713-30-1-225 20806452

[15] Conn HJ Taxonomic relationships of certain non-sporeforming rods in soil. J Bacteriol 1938; 36:320-321

[16] Frank B Über die Pilzsymbiose der Leguminosen. Ber Dtsch Bot Ges 1889; 7:332-346

[17] Jordan DC, Allen ON. Genus I. *Rhizobium* Frank 1889, 338; Nom. gen. cons. Opin. 34, Jud. Comm. 1970, 11. In: Buchanan RE, Gibbons NE (eds), Bergey's Manual of Determinative Bacteriology, Eighth Edition, The Williams and Wilkins Co., Baltimore, 1974, p. 262-264.

[18] Young JM Kuykendall LD Martínez-Romero E Kerr A Sawada H A revision of *Rhizobium* Frank 1889, with an emended description of the genus, and the inclusion of all species of *Agrobacterium* Conn 1942 and *Allorhizobium undicola* de Lajudie et al. 1998 as new combinations: *Rhizobium radiobacter, R. rhizogenes, R. rubi, R. undicola* and *R. vitis.* Int J Syst Evol Microbiol 2001; 51:89-103 11211278 10.1099/00207713-51-1-89

[19] Editorial Secretary (for the Judicial Commission of the International Committee on Nomenclature of Bacteria). OPINION 34: Conservation of the Generic Name *Rhizobium* Frank 1889. Int J Syst Bacteriol 1970; 20:11-12 10.1099/00207713-20-1-11

[20] Ramírez-Bahena MH García-Fraile P Peix A Valverde A Rivas R Igual JM Mateos PF Martínez-Molina E Velázquez E Revision of the taxonomic status of the species *Rhizobium leguminosarum* (Frank 1879) Frank 1889AL, *Rhizobium phaseoli* Dangeard 1926AL and *Rhizobium trifolii* Dangeard 1926AL. *R. trifolii* is a later synonym of *R. leguminosarum*. Reclassification of the strain *R. leguminosarum* DSM 30132 (=NCIMB 11478) as *Rhizobium pisi* sp. nov. Int J Syst Evol Microbiol 2008; 58:2484-2490 10.1099/ijs.0.65621-0 18984681

[21] Agents B. Technical rules for biological agents. TRBA (http://www.baua.de):466.

[22] Ashburner M Ball CA Blake JA Botstein D Butler H Cherry JM Davis AP Dolinski K Dwight SS Eppig JT Gene ontology: tool for the unification of biology. The Gene Ontology Consortium. Nat Genet 2000; 25:25-29 10.1038/75556 10802651 PMC3037419

[23] Tamura K Peterson D Peterson N Stecher G Nei M Kumar S MEGA5: molecular evolutionary genetics analysis using maximum likelihood, evolutionary distance, and maximum parsimony methods. Mol Biol Evol 2011; 28:2731-2739 10.1093/molbev/msr121 21546353 PMC3203626

[24] Felsenstein J Confidence limits on phylogenies: an approach using the bootstrap. Evolution 1985; 39:783-791 10.2307/2408678 28561359

[25] Liolios K Mavromatis K Tavernarakis N Kyrpides NC The Genomes On Line Database (GOLD) in 2007: status of genomic and metagenomic projects and their associated metadata. Nucleic Acids Res 2008; 36:D475-D479 10.1093/nar/gkm884 17981842 PMC2238992

[26] Reeve WG Tiwari RP Worsley PS Dilworth MJ Glenn AR Howieson JG Constructs for insertional mutagenesis, transcriptional signal localization and gene regulation studies in root nodule and other bacteria. Microbiology 1999; 145:1307-1316 10.1099/13500872-145-6-1307 10411257

[27] DOE Joint Geonme Institute http://my.jgi.doe.gov/general/index.html

[28] Bennett S Solexa Ltd. Pharmacogenomics 2004; 5:433-438 10.1517/14622416.5.4.433 15165179

[29] Margulies M Egholm M Altman WE Attiya S Bader JS Bemben LA Berka J Braverman MS Chen YJ Chen Z Genome sequencing in microfabricated high-density picolitre reactors. Nature 2005; 437:376-380 16056220 10.1038/nature03959 PMC1464427

[30] DOE Joint Genome Institute http://my.jgi.doe.gov/general/index.html

[31] Zerbino DR. Using the Velvet *de novo* assembler for short-read sequencing technologies. Current Protocols in Bioinformatics 2010;Chapter 11:Unit 11 5. 10.1002/0471250953.bi1105s31 PMC2952100 20836074

[32] Hyatt D Chen GL Locascio PF Land ML Larimer FW Hauser LJ Prodigal: prokaryotic gene recognition and translation initiation site identification. BMC Bioinformatics 2010; 11:119 10.1186/1471-2105-11-119 20211023 PMC2848648

[33] Mavromatis K Ivanova NN Chen IM Szeto E Markowitz VM Kyrpides NC The DOE-JGI Standard operating procedure for the annotations of microbial genomes. Stand Genomic Sci 2009; 1:63-67 10.4056/sigs.632 21304638 PMC3035208

[34] Pati A Ivanova NN Mikhailova N Ovchinnikova G Hooper SD Lykidis A Kyrpides NC GenePRIMP: a gene prediction improvement pipeline for prokaryotic genomes. Nat Methods 2010; 7:455-457 10.1038/nmeth.1457 20436475

[35] Lowe TM Eddy SR tRNAscan-SE: a program for improved detection of transfer RNA genes in genomic sequence. Nucleic Acids Res 1997; 25:955-964 9023104 10.1093/nar/25.5.955 PMC146525

[36] Lagesen K Hallin P Rodland EA Staerfeldt HH Rognes T Ussery DW RNAmmer: consistent and rapid annotation of ribosomal RNA genes. Nucleic Acids Res 2007; 35:3100-3108 10.1093/nar/gkm160 17452365 PMC1888812

[37] Griffiths-Jones S Bateman A Marshall M Khanna A Eddy SR Rfam: an RNA family database. Nucleic Acids Res 2003; 31:439-441 10.1093/nar/gkg006 12520045 PMC165453

[38] Krogh A Larsson B von Heijne G Sonnhammer EL Predicting transmembrane protein topology with a hidden Markov model: application to complete genomes. J Mol Biol 2001; 305:567-580 10.1006/jmbi.2000.4315 11152613

[39] Bendtsen JD Nielsen H von Heijne G Brunak S Improved prediction of signal peptides: SignalP 3.0. J Mol Biol 2004; 340:783-795 10.1016/j.jmb.2004.05.028 15223320

[40] Markowitz VM Mavromatis K Ivanova NN Chen IM Chu K Kyrpides NC IMG ER: a system for microbial genome annotation expert review and curation. Bioinformatics 2009; 25:2271-2278 10.1093/bioinformatics/btp393 19561336

